# Environmental Application of Newly Designed Loop-Mediated Isothermal Amplification (LAMP) Kits for Nosocomial Pathogen Detection in Hospital Settings

**DOI:** 10.3390/life16060994

**Published:** 2026-06-12

**Authors:** Federica Marino, Caterina Bonincontro, Laura Caligaris, Carlo Derelitto, Luna Girolamini, Sandra Cristino

**Affiliations:** Department of Biological, Geological, and Environmental Sciences, University of Bologna, 40126 Bologna, Italycaterina.bonincontr2@unibo.it (C.B.); laura.caligaris2@unibo.it (L.C.); carlo.derelitto@unibo.it (C.D.); luna.girolamini2@unibo.it (L.G.)

**Keywords:** loop-mediated isothermal amplification, healthcare-associated infections, nosocomial pathogens, environmental monitoring, hospital surfaces

## Abstract

Nosocomial pathogens persist on hospital surfaces contributing to healthcare-associated infections (HAIs), especially among vulnerable patients and in the presence of multidrug-resistant strains. Environmental surveillance is essential to prevent cross-contamination and support timely infection control interventions. However, conventional culture-based methods, although considered the gold standard, are labor-intensive and time-consuming, often delaying critical responses. This study evaluated loop-mediated isothermal amplification (LAMP) as a rapid screening tool for hospital environmental monitoring. A total of 100 surface samples were collected from different hospital wards and analyzed using both culture and LAMP assays targeting six major HAI-related pathogens: *Pseudomonas aeruginosa*, *Staphylococcus aureus*, *Enterococcus* spp., *Escherichia coli*, *Klebsiella pneumoniae*, and *Acinetobacter baumannii*. LAMP showed excellent performance, with sensitivity of 1.00 for all targets and specificity ranging from 0.94 to 1.00. No statistically significant differences were observed between LAMP and culture results (*p* > 0.05). LAMP may represent a valuable complementary method for routine environmental surveillance.

## 1. Introduction

Nosocomial pathogens are defined as microorganisms capable of causing infections in patients during hospitalization or medical treatment, commonly referred to as healthcare-associated infections (HAIs) [1]. These infections, which are neither present nor incubating at the time of hospital admission, represent a serious threat to patient safety and public health, as they are associated with increased morbidity, mortality, and healthcare costs. Globally, it is estimated that between 5% and 15% of hospitalized patients are affected by HAIs [2]. In Europe alone, approximately 2.6 million new cases of HAIs are reported annually [3], with an estimated 37,000 deaths directly attributable to these infections and a further 110,000 cases in which HAIs act as a contributing factor [4]. These infections typically originate from exposure to healthcare environments, underscoring the critical importance of hospital environmental surveillance in preventing their occurrence [5].

According to the most recent data from the ECDC, the most frequently isolated pathogens in HAIs across acute care hospitals include: *Escherichia coli* (*E. coli*, 12.7%), *Klebsiella pneumoniae* (*K. pneumoniae*, 11.7%), *Enterococcus* spp. (10.0%), *Staphylococcus aureus* (*S. aureus*, 9.0%), *Pseudomonas aeruginosa* (*P. aeruginosa*, 7.9%), and *Acinetobacter* spp. (3.2%) [6]. These pathogens are responsible for a wide range of clinical conditions, including urinary tract infections, bloodstream infections, surgical site infections, and ventilator-associated pneumonia [7]. They are often highly resistant to both disinfectants and antibiotics, making them particularly difficult to eliminate. Their persistence in hospital environments, especially on surfaces where they can form biofilms, increases the risk of transmission among patients and healthcare workers, facilitating their spread within healthcare facilities [8,9]. This resistance poses significant challenges for clinical management and infection prevention, underscoring the need for stringent hygiene protocols, continuous environmental surveillance, and the adoption of innovative diagnostic approaches to promptly detect and contain nosocomial pathogens.

Conventional methods for assessing microbial contamination on hospital surfaces typically involve the use of agar contact plates or swabs, followed by culture-based technique, which is considered the gold standard [10,11,12]. After sample collection, this method requires extended incubation periods to allow colony growth. Subsequent identification of the isolates (often through biochemical or mass spectrometry-based approaches) further prolongs the analysis timeline, delaying prompt intervention [13]. This delay can pose a significant challenge, especially in healthcare settings where vulnerable patients are at increased risk of infection and where multidrug-resistant strains are becoming increasingly prevalent in the environment [14,15]. Rapid and reliable detection methods are therefore essential to support prompt infection control measures and minimize the risk of nosocomial outbreaks.

In recent years, molecular techniques have been increasingly explored as complementary tools to overcome some of the constraints of growth-based methods [16]. Among these, quantitative PCR (qPCR) remains the most widely adopted molecular approach for pathogen detection due to its high analytical sensitivity and specificity. However, its routine implementation in hospital environmental surveillance may be limited by workflow complexity, infrastructure requirements, and turnaround time, particularly when frequent or large-scale surface monitoring is required [17] (Alsharksi et al., 2024).

In this context, loop-mediated isothermal amplification (LAMP) has emerged as a promising alternative, offering fast and sensitive detection of microbial DNA while relying on simpler instrumentation and more streamlined workflows [18]. This technique enables the rapid and specific detection of target DNA sequences under isothermal conditions, typically at 60–65 °C, thereby eliminating the need for a thermocycler and allowing for simpler and time-efficient workflows [19,20]. The method relies on a set of four to six primers tailored to recognize six distinct regions on the target DNA [21]. This multi-primer architecture markedly enhances assay specificity, as the amplification process requires the coordinated hybridization of multiple primers to spatially distinct regions of the target sequence. This multi-site recognition imposes stringent sequence requirements, significantly reducing the likelihood of non-specific amplification events and off-target binding [19,22,23]. These primers initiate the amplification reaction, which is carried out by a DNA polymerase with high strand-displacement activity [24]. This enzymatic property enables the polymerase to synthesize new DNA strands while simultaneously displacing existing ones, allowing for continuous amplification under isothermal conditions [25]. As the reaction progresses, loop structures are formed, further accelerating the process and resulting in the accumulation of large amounts of DNA within 30 to 60 min [26]. Amplification can be detected in real-time or at the endpoint using various methods, including turbidity (resulting from magnesium pyrophosphate precipitation), fluorescence (with intercalating dyes), or colorimetric changes (utilizing pH-sensitive indicators) [27].

Thanks to its simplicity, speed, and versatility, LAMP has already been successfully applied in a variety of fields, ranging from clinical diagnostics to food safety, veterinary medicine, and environmental monitoring of water [28,29,30,31].

Additionally, beyond these established contexts, our research has focused on adapting LAMP technology to hospital environmental surveillance. In this framework, our previous studies evaluated the feasibility of applying LAMP assays originally developed for water analysis to surface monitoring, under controlled and preliminary real-world conditions [32,33]. These studies demonstrated methodological transferability and supported the potential of LAMP for environmental applications, providing the basis for further technological development.

Building on this experience, the present study represents a subsequent and distinct step. Specifically, it evaluates newly developed commercial LAMP kits explicitly designed for hospital surface monitoring, targeting a broader and clinically relevant panel of nosocomial pathogens (*P. aeruginosa*, *S. aureus*, *Enterococcus* spp., *E. coli*, *K. pneumoniae*, and *A. baumannii*) [34].

Unlike most LAMP studies conducted under controlled conditions or focused on single-pathogen detection, this work applies LAMP within a real-world hospital surveillance setting. Its novelty lies in the translation of LAMP into a practical, surveillance-oriented tool for routine environmental monitoring. The assays were applied under routine hospital surveillance conditions and systematically compared with the gold-standard culture-based method.

The aim of this work was therefore not to replace conventional culture techniques, but to assess the applicability, performance, and operational value of LAMP as a rapid screening tool within hospital environmental surveillance workflows. By enabling same-day results and supporting timely decision-making, LAMP may enhance risk assessment, guide sanitation strategies, and complement established microbiological surveillance practices.

## 2. Materials and Methods

A total of 100 surface samples were collected from various hospital environments, including operating rooms, intensive care units, patient rooms, and common areas, as part of an environmental surveillance campaign conducted in an Italian hospital from July to September 2025. All samples were analyzed using both the conventional culture-based method, considered the gold standard, and the LAMP technique for rapid DNA detection.

### 2.1. Sample Collection

Sampling was performed using a kit developed and provided by Enbiotech (Palermo, Italy), which includes sterile swabs, a neutralizing solution, and sterile tubes pre-filled with an enrichment broth. The exact composition of the reagents and media is protected under the manufacturer’s patent.

To maximize microbial recovery, each swab was first moistened in the neutralizing solution and then passed over the target surface in three directions (vertical, horizontal, and diagonal) while applying uniform pressure and rotating the swab. After sampling, the swab was immediately placed into the sterile tube preloaded with enrichment broth (3 mL) and stored at 4 °C for no longer than ten hours before analysis.

In accordance with UNI EN 17141:2021, for flat surfaces, a standardized area of 100 cm^2^ was sampled using sterile templates to precisely delimit the sampling zone [35]. For irregular surfaces, the sampled area was estimated using a previously sterilized measuring tape.

All sampling and handling procedures were standardized across all sites to minimize variability and ensure reproducibility.

### 2.2. Culture-Based Method

For culture-based analysis, an aliquot of 200 µL of each swab enrichment broth (used as the transport medium) was plated onto tryptic soy agar (TSA) plates (Thermo Fisher Scientific, Diagnostic, Ltd., Basingstoke, UK) and incubated at 35 ± 2 °C for 72 h. Following incubation, all visible colonies were counted and differentiated based on their morphological characteristics. Identification was performed using the Matrix-Assisted Laser Desorption/Ionization—Time of Flight (MALDI) Biotyper^®^ (Bruker Daltonics, Bremen, Germany).

Results were expressed as colony-forming units (CFU) per 200 µL (CFU/200 µL), representing the number of viable microorganisms recovered from each sampled surface. For comparative purposes, culture-based results were subsequently categorized dichotomously as positive or negative based on the recovery of target microorganisms on selective or semi-selective media. This binary classification allowed direct comparability with the qualitative LAMP results.

The culture method, under the applied experimental conditions, theoretically allowed detection of at least 1 CFU in the plated 200 µL sample volume, considered indicative of a positive result.

### 2.3. LAMP Analysis

All swabs immersed in the remaining 2.8 mL of enrichment broth were incubated at 35 ± 2 °C for 6 h. After incubation, samples were stored at 4 °C for up to 24 h before analysis.

DNA extraction was performed using reagents and protocols provided by the LAMP kit manufacturer (Enbiotech, Palermo, Italy). Specifically, for each swab, a 1.8 mL aliquot of the enriched broth was transferred into sterile 2 mL tubes included in the kit, which were then processed according to the manufacturer’s DNA extraction protocol.

In detail, the samples were centrifuged at 9500× *g* for 5 min, and the supernatant was discarded by inversion. The resulting pellet was resuspended in 200 µL of TE 1X buffer, and the tubes were incubated at 95 °C for 10 min using a dry block heater. Subsequently, the samples were centrifuged at maximum speed for 20 s, and the supernatant containing the extracted DNA was used as the template for the amplification. Following DNA extraction, each environmental sample was tested for all six target pathogens using separate, pathogen-specific LAMP assays, allowing multiple microorganisms to be screened from the same swab in parallel, resulting in a total of 600 individual reactions across the study. Each target pathogen was analyzed in a separate LAMP reaction using pathogen-specific primer sets, thereby minimizing the risk of cross-reactivity and non-specific amplification. The extracted DNA from each sample was used as a template for six separate amplification reactions, each performed with a different LAMP kit targeting *P. aeruginosa*, *S. aureus*, *Enterococcus* spp., *E. coli*, *K. pneumoniae*, and *A. baumannii*, all provided by Enbiotech (Palermo, Italy). Each reaction tube was prepared as follows:

- 14 µL of LAMP Mix and 6 µL of DNA for *P. aeruginosa*, *S. aureus*, *E. coli*, *K. pneumoniae*, and *A. baumannii*;
- 19 µL of LAMP Mix and 6 µL of DNA for *Enterococcus* spp.

The DNA templates were carefully pipetted to ensure proper rehydration and activation of the lyophilized primers located at the bottom of each reaction tube. All samples were then loaded into the ICGENE Plus instrument (Enbiotech, Palermo, Italy) for amplification. All LAMP runs included manufacturer-provided positive and negative controls, which were systematically processed in each session to ensure assay performance and to exclude contamination or non-specific amplification. All reagents used in the amplification process are covered by the intellectual property of the manufacturer.

Results were visualized in real time using the ICGENE Application (version 3.9.10), installed on a dedicated tablet supplied with the instrument, which automatically interprets the outcome of each reaction and displays the result as either positive (‘+’) or negative (‘–’). The limit of detection (LoD) declared by the manufacturer for the LAMP assays used in this study ranges from 0.2 to 2 CFU per sample, depending on the target pathogen. LAMP results were interpreted according to the manufacturer-validated qualitative output and reported as presence or absence of the target microorganism. Samples were considered positive when a positive signal (‘+’) was automatically generated by the system, and negative otherwise.

To confirm the results obtained through LAMP analysis and recover the isolates for further characterization, a 100 µL aliquot of the remaining enrichment broth was plated onto both selective and semi-selective media following the incubation period, in order to verify the presence or absence of the target microorganisms. The following selective culture media were used to recover specific microorganisms: Cetrimide agar for *P. aeruginosa*, Baird-Parker agar for *S. aureus*, Slanetz agar for *Enterococcus* spp., Coliform Count agar for *E. coli*, and MacConkey agar for both *K. pneumoniae* and *A. baumannii*. All media used were provided by Thermo Fisher Scientific, Diagnostic, Ltd. (Basingstoke, UK). Culture results were considered positive when growth of the target microorganism was observed on the appropriate selective or semi-selective medium, and negative in the absence of detectable growth.

To minimize observational bias, LAMP and culture-based analyses were performed independently. The interpretation of results for each method was conducted without prior knowledge of the outcome obtained with the other technique.

### 2.4. Data Analysis

The data analysis was conducted using R software (version 4.5.1), starting from contingency tables constructed by comparing the results obtained with the experimental LAMP assays to those from the culture-based method, used as the reference (R Core Team, 2024). To ensure comparability between methods, culture-based results were also categorized as either positive or negative. For performance evaluation, samples were classified based on LAMP–culture concordance: matches between positive results were considered true positives, LAMP-positive/culture-negative results as false positives, concordant negatives as true negatives, and LAMP-negative/culture-positive results as false negatives. The complete contingency tables for each pathogen are provided in the Supplementary Materials (Tables S1–S6).

To assess the analytical performance of the LAMP method, a comprehensive set of statistical metrics was calculated for each kit, including sensitivity (Se), specificity (Sp), with their respective 95% confidence intervals (95% CI), positive predictive value (PPV), negative predictive value (NPV), accuracy (Acc), F1-score (F1), balanced accuracy (Bal Acc), and Youden’s index (J) [36,37]. To further strengthen the comparative analysis, McNemar’s test was used to evaluate the significance of discordant classifications between the LAMP and culture-based methods, providing insight into potential systematic biases [38].

Finally, to better quantify the level of agreement between the LAMP and culture-based methods, the prevalence-adjusted bias-adjusted kappa (PABAK) coefficient was calculated. PABAK adjusts for both prevalence and bias, providing a more reliable and interpretable measure of concordance when the dataset has an unequal distribution of positive and negative results [39].

## 3. Results

**Figure 1:**
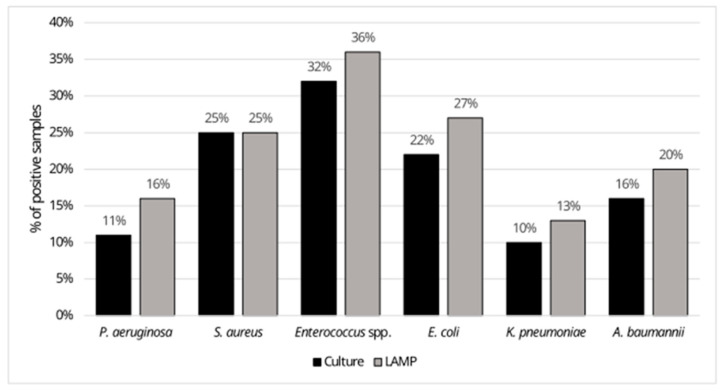
Positive sample detection rates (%) by culture-based methods and LAMP for the six investigated pathogens. Bars represent the proportion of positive samples detected by each method.

To evaluate the analytical performance of the experimental approach, a comparative analysis was conducted between the results obtained with the LAMP assays and those from the culture-based method for each pathogen. The percentages of positive samples detected by each method were assessed, highlighting potential differences in detection rates (Figure 1).

The results showed that, out of a total of 100 surface samples, *Enterococcus* spp. were the most frequently detected pathogens by both methods (culture: 32%; LAMP: 36%), while *K. pneumoniae* was the least frequently identified (culture: 10%; LAMP: 13%). For *S. aureus*, both methods yielded the same detection rate (25%), while for the remaining pathogens, LAMP detected a higher percentage of positive samples compared to the culture-based method (e.g., *P. aeruginosa*: 16% vs. 11%; *E. coli*: 27% vs. 22%; *A. baumannii*: 20% vs. 16%).

Following the comparison of detection frequencies, a set of performance metrics was calculated for all the LAMP kits to evaluate the analytical capability of the innovative method, using the culture-based technique as the reference standard (Table 1).

Specifically, Se values were equal to 1.00 for all target pathogens, while Sp ranged from a minimum of 0.94 for *P. aeruginosa*, *Enterococcus* spp., and *E. coli* to a maximum of 1.00 for *S. aureus*. Overall, the *S. aureus* kit exhibited the highest performance, achieving maximum values across all evaluated indicators. In contrast, the *P. aeruginosa* kit showed the lowest values, particularly in terms of PPV and F1.

Acc, NPV, Bal Acc, and J remained high across all tested kits. The level of agreement between the LAMP method and the reference culture-based method was assessed using the PABAK, which indicated strong concordance for all kits, with values ranging from 0.90 to 1.00.

The statistical comparison between the LAMP method and the gold-standard method was further assessed using the McNemar test, applied to paired nominal data. The test was performed to evaluate whether significant differences existed in the classification outcomes between the two methods (Table 2).

No statistically significant differences were observed between the two methods (*p* > 0.05) for any of the pathogens tested. For *S. aureus*, the test could not be performed due to the absence of discordant pairs.

## 4. Discussion

In recent years, the prevention of HAIs has become an increasingly critical issue worldwide, particularly due to the rising prevalence of multidrug-resistant microorganisms [40]. Scientific evidence has demonstrated that pathogenic bacteria are capable of persisting in hospital environments for extended periods (from days to months), significantly increasing the risk of cross-contamination [41].

Within this context, rapid environmental monitoring plays a crucial role in enabling more effective control of microbial contamination and limiting the spread of harmful agents. For this reason, the use of LAMP technology—already proven to yield promising results in recent studies [42,43,44]—could represent a valuable tool when applied to hospital environmental surveillance. Indeed, its ability to provide fast and reliable detection may substantially reduce response times and enhance the overall safety of healthcare settings.

Building on this premise, the present study represents the final phase of a broader research project that initially focused on adapting LAMP kits originally developed for water analysis to the monitoring of hospital surfaces [32]. After demonstrating that these kits could achieve reliable performance under both laboratory-controlled conditions and real-world hospital settings [33], the research advanced toward the development and validation of specialized kits targeting the pathogens most frequently associated with HAIs. This article presents the results of this final phase, focusing on the analytical performance of the newly developed LAMP kits when applied “in real conditions” to hospital surface samples monitoring. These efforts aimed to further optimize the applicability of LAMP for routine environmental surveillance in hospital facilities. Importantly, these results were obtained under real environmental conditions, reflecting the performance of the method in routine hospital surveillance rather than under controlled laboratory settings.

Overall, the findings of this study confirm the high analytical performance of LAMP in detecting nosocomial pathogens on hospital surfaces. Notably, all six tested kits achieved a maximum Se of 1.00, indicating their ability to correctly identify all true positive samples. Sp values were also high, ranging from 0.94 (for *P. aeruginosa*, *Enterococcus* spp., and *E. coli*) to 1.00 (for *S. aureus*), demonstrating strong discriminatory power. These results are consistent with those reported by 32 and align with other recent studies evaluating LAMP as a rapid and reliable diagnostic tool for microbial detection in both clinical and environmental contexts [27,45].

In particular, the high Se values clearly reflect the superior capability of LAMP to detect positive samples compared to traditional culture-based methods. Several comparative studies have demonstrated that molecular methods consistently detect a greater number of positive samples than culture-based techniques. For example, Alsharksi et al. [17] and Váradi et al. [46] emphasize the enhanced sensitivity of molecular diagnostics over conventional approaches, especially in complex or low-biomass samples. This discrepancy can be attributed to several methodological and biological factors. First, molecular techniques like LAMP are capable of detecting bacterial DNA regardless of cell viability, allowing the detection of pathogens even when the cells are no longer alive [47]. In the context of environmental monitoring, this is not a major limitation, as false positives may simply prompt precautionary measures (e.g., and addinional disinfection of surface, etc.), without compromising safety, while missed detections could pose a greater risk to infection control. Second, LAMP, as a molecular method, can detect viable but non-culturable (VBNC) cells—bacteria that are metabolically active and potentially infectious but fail to grow under standard laboratory conditions [48,49]. This is particularly relevant in hospital settings, where environmental stressors such as disinfectants or nutrient limitations may induce a VBNC state, potentially leading to false-negative outcomes in culture-based assessments [50].

For this reason, the pre-enrichment step was included to promote recovery of viable bacterial cells and increase the probability of detecting low-abundance or sub-lethally injured microorganisms prior to LAMP analysis.

Although pre-enrichment may introduce certain limitations, such as extending the overall analysis time compared to direct molecular detection, in the context of environmental surveillance this step is essential to ensure that detected signals are associated with viable organisms rather than residual nucleic acids. Moreover, unlike conventional enrichment periods (typically 18–24 h), the protocol applied here employed a shortened 6 h pre-enrichment phase previously optimized to improve turnaround time while maintaining microbial recovery, allowing same-day results and maintaining a substantially faster workflow compared to culture-based methods.

Moreover, although international standards such as UNI EN 17141:2021 recommend the use of non-selective media like TSA for environmental monitoring, its non-selective nature poses significant challenges in interpreting results, particularly in environments with high microbial loads. In such cases, multiple species may grow simultaneously, leading to overlapping colonies and complex morphologies that are difficult to differentiate and identify [51]. Accurate identification often depends on the operator’s experience and subjective judgment, which introduces variability and potential for misclassification, especially when colonies obscure one another or exhibit atypical growth patterns. These interpretative challenges highlight the limitations of relying solely on non-selective culture media for environmental monitoring and underscore the value of integrating molecular methods such as LAMP, which offer species-specific detection and reduce operator-dependent variability.

To mitigate these limitations, the present study supplemented TSA with the use of selective media tailored to the target pathogens. Selective media inhibit the growth of non-target organisms and enhance the visibility of specific colony types, thereby improving the accuracy of identification. This dual approach—combining broad-spectrum and targeted cultivation—was designed to strengthen the reliability of microbiological assessments and enable a more meaningful comparison with molecular detection outcomes. Thanks to the adoption of this approach, *Enterococcus* spp. emerged as the most frequently isolated pathogen, in line with previous studies highlighting its prevalence in the environment thanks to its ability to survive under adverse conditions [52,53].

Among the tested kits, the *S. aureus* kit demonstrated perfect agreement across all metrics, confirming its robustness and reliability. In contrast, the *P. aeruginosa* kit showed comparatively lower PPV (0.69) and F1-score (0.81). This may be attributed to the known challenges in recovering *P. aeruginosa* from surface samples using culture-based methods, as previously reported in the literature [54]. Its poor cultivability in environmental matrices, possibly linked to biofilm formation and stress-induced dormancy, could lead to underestimation in traditional assessments, thereby affecting comparative performance metrics.

Beyond these two cases, the kits targeting *Enterococcus* spp., *E. coli*, *K. pneumoniae*, and *A. baumannii* showed Bal Acc values above 0.97 and PABAK scores between 0.90 and 0.96, confirming strong concordance with the reference method and supporting their reliability for routine environmental surveillance.

Moreover, the McNemar test did not reveal statistically significant differences between LAMP and culture-based methods for any pathogen (*p* > 0.05), further supporting the equivalence of the two approaches in terms of classification accuracy. For *S. aureus*, the test could not be performed due to the absence of discordant pairs, reflecting perfect agreement between methods. This is particularly relevant given the limitations of culture, which is time-consuming and may fail to detect stressed or low-abundance organisms [55].

These findings align with a growing body of literature on molecular diagnostics, which increasingly favors LAMP for its speed, sensitivity, and adaptability to field conditions [56,57]. In this study, the rapidity of LAMP is further demonstrated by its ability to detect six different pathogens from a single surface sample, following a single extraction process. This capability significantly streamlines the workflow and enhances the efficiency of environmental surveillance, making LAMP a highly practical tool for routine hospital monitoring. This multi-target approach represents a practical advantage for environmental surveillance, where rapid and comprehensive screening is required. Unlike most LAMP studies focused on single-pathogen detection, this study demonstrates the feasibility of multi-pathogen screening within a single workflow, addressing the complexity of hospital environments.

However, the use of molecular methods such as LAMP cannot entirely replace traditional culture-based techniques, which remain essential for pathogen isolation and epidemiological investigation. Moreover, culturing the strain is fundamental for conducting antimicrobial resistance profiling and for comparing environmental isolates with clinical strains to trace sources of contamination and infection within healthcare settings during epidemiological investigation. According to the directives and guidelines the “real risk” is associated with the viable cells, for this reason other than for the questions above mentioned the culture technique was the only technique used as reference in this study. Nevertheless, due to the inherent limitations of culture-based methods—such as long incubation times, reduced sensitivity in detecting VBNC organisms, and challenges in interpreting mixed microbial growth—they are not sufficient on their own to ensure effective monitoring and prevention. For this reason, integrating rapid and easy-to-perform molecular techniques like LAMP alongside conventional methods can significantly enhance the overall surveillance strategy. One limitation of this study is the absence of further confirmation of positive LAMP test results via qPCR and sequencing. However, this is offset by the use of a culture technique, which is essential for assessing the actual risk posed by the presence of pathogens on the analysed surface. The ability to perform rapid disinfection based on positive LAMP test results does not increase the time or workload for operators, thereby mitigating the issue of false-negative culture results.

Beyond analytical comparison, these findings support a surveillance-oriented framework in which LAMP can be positioned upstream of culture-based methods as a rapid screening step. This approach enables same-day identification of potentially contaminated surfaces, while culture remains essential for isolate recovery and downstream analyses.

## 5. Conclusions

The present study demonstrated the applicability and performance of LAMP-based screening under routine hospital surveillance conditions. While further studies, including multicenter evaluations, will be required to extend the generalizability of these findings, the results obtained here support the integration of LAMP as a complementary approach within established culture-based environmental surveillance workflows.

Indeed, its speed and simplicity make it particularly effective for preliminary screening before culture, allowing laboratories to prioritize samples and optimize resources. Furthermore, this approach could facilitate the rapid revalidation of sanitized areas, providing timely confirmation of cleaning efficacy procedures. By complementing culture-based methods with LAMP, healthcare facilities can adopt a more proactive and responsive approach to infection prevention, improving both the speed and accuracy of environmental monitoring.

## Figures and Tables

**Table 1:** Performance metrics calculated for each LAMP kit. Sensitivity (Se), specificity (Sp), with 95% CI accuracy (Acc), positive predictive value (PPV), negative predictive value (NPV), F1-score (F1), and balanced accuracy (Bal Acc).

LAMP Kit	Se	95% CI	Sp	95% CI	Acc	PPV	NPV	F1	Bal Acc	J	PABAK
*P. aeruginosa*	1.00	(0.72–1.00)	0.94	(0.87–0.98)	0.95	0.69	1.00	0.81	0.97	0.94	0.90
*S. aureus*	1.00	(0.86–1.00)	1.00	(0.95–1.00)	1.00	1.00	1.00	1.00	1.00	1.00	1.00
*Enterococcus* spp.	1.00	(0.89–1.00)	0.94	(0.85–0.98)	0.96	0.89	1.00	0.94	0.97	0.94	0.92
*E. coli*	1.00	(0.85–1.00)	0.94	(0.86–0.97)	0.95	0.81	1.00	0.90	0.97	0.94	0.90
*K. pneumoniae*	1.00	(0.69–1.00)	0.97	(0.91–0.99)	0.97	0.77	1.00	0.87	0.98	0.97	0.94
*A. baumannii*	1.00	(0.79–1.00)	0.96	(0.88–0.99)	0.96	0.80	1.00	0.89	0.98	0.96	0.92

**Table 2:** The McNemar test results comparing the LAMP method with the reference culture-based method.

Pathogen Detected	*p*-Value
*P. aeruginosa*	0.074
*S. aureus*	NA
*Enterococcus* spp.	0.134
*E. coli*	0.074
*K. pneumoniae*	0.248
*A. baumannii*	0.134

## Data Availability

All relevant data are provided in the manuscript. The corresponding author can provide additional data as a reasonable request.

## Supplementary Materials

The following supporting information can be downloaded at: https://www.mdpi.com/article/10.3390/life16060994/s1, Table S1: 2 × 2 continegency table for *Pseudomonas aeruginosa*; Table S2: 2 × 2 continegency table for *Staphylococcus aureus*; Table S3: 2 × 2 continegency table for *Enterococcus* spp.; Table S4: 2 × 2 continegency table for *Escherichia coli*; Table S5: 2 × 2 continegency table for *Klebsiella pneumoniae*; Table S6: 2 × 2 continegency table for *Acinetobacter baumannii*.
