# Supplementary material for: Environmental Application of Newly Designed Loop-Mediated Isothermal Amplification (LAMP) Kits for Nosocomial Pathogen Detection in Hospital Settings
Source: Life (Basel). 2026 Jun 12;16(6):994. doi: 10.3390/life16060994 (PMC13300737; doi:10.3390/life16060994)
Supplement: Supplementary file 1 [file life-16-00994-s001.zip › life-4279880-supplementary.pdf]

SUPPLEMENTARY MATERIAL

P. aeruginosa		**Gold standard**	
		+	-
**LAMP**	+	11	5
	-	0	84

Table S1. 2×2 contingency table for *Pseudomonas aeruginosa.*

S. aureus		**Gold standard**	
		+	-
**LAMP**	+	25	0
	-	0	75

Table S2. 2×2 contingency table for *Staphylococcus aureus.*

***Enterococcus* spp.**		**Gold standard**	
		+	-
**LAMP**	+	32	4
	-	0	64

Table S3. 2×2 contingency table for *Enterococcus spp.*

E. coli		**Gold standard**	
		+	-
**LAMP**	+	22	5
	-	0	73

Table S4. 2×2 contingency table for *Escherichia coli.*

K. pneumoniae		**Gold standard**	
		**+**	**-**
**LAMP**	**+**	10	3
	**-**	0	87

Table S5. 2×2 contingency table for *Klebsiella pneumoniae.*

A. baumannii		**Gold standard**	
		**+**	**-**
**LAMP**	**+**	16	4
	**-**	0	80

Table S6. 2×2 contingency table for *Acinetobacter baumannii.*
